# Supplementary material for: Experience in different modes of delivery in twin pregnancy
Source: PLoS One. 2022 Mar 11;17(3):e0265180. doi: 10.1371/journal.pone.0265180 (PMC8916635; doi:10.1371/journal.pone.0265180)
Supplement: Attachment — Submitted filename: Responce to reviewers.docx [file pone.0265180.s002.docx]

Nov, 9, 2021

RE: Manuscript ID PONE-D-21-17824 entitled “Experience in Different Modes of Delivery in Twin Pregnancy”

Dear Editorial Board,

Thank you for your letter on 9 Oct 2021. I am resubmitting my revised manuscript entitled “Experience in Different Modes of Delivery in Twin Pregnancy.”

In this revised manuscript, I have made corrections follow reviewer A’s comments. Attached please also find my response to reviewer A’s comment, which is very helpful to improve this manuscript. Please kindly express my sincere gratefulness to the peer-reviewers.

I am looking forward to hearing from your decision.

Yours sincerely,

Pao-Ling Torng, MD, PhD

Department of Obstetrics and Gynecology, National Taiwan University Hospital, No. 7, Chung Shan South Road, Taipei 10002, Taiwan.

Email: pltorng@ntu.edu.tw.

Tel: 886-223123456 ext. 71558

Fax: 886-223114965

Journal Requirements:

*1. Please ensure that your manuscript meets PLOS ONE's style requirements, including those for file naming. The PLOS ONE style templates can be found at*

*https://journals.plos.org/plosone/s/file?id=wjVg/PLOSOne_formatting_sample_main_body.pdf and* [*https://journals.plos.org/plosone/s/file?id=ba62/PLOSOne_formatting_sample_title_authors_affiliations.pdf*](https://journals.plos.org/plosone/s/file?id=ba62/PLOSOne_formatting_sample_title_authors_affiliations.pdf)

**Response:** We have checked our manuscript to meet the PLOS ONE’s style requirements.

*2. Thank you for including your ethics statement: "The ethical committees of National Taiwan University Hospital, Hsin-Chu Branch (reference no. 108-119-E)"*

*a) Please amend your current ethics statement to include the full name of the ethics committee/institutional review board(s) that approved your specific study.*

*b) Please provide additional details regarding participant consent. In the ethics statement in the Methods and online submission information, please ensure that you have specified (1) whether consent was informed and (2) what type you obtained (for instance, written or verbal, and if verbal, how it was documented and witnessed). If your study included minors, state whether you obtained consent from parents or guardians. If the need for consent was waived by the ethics committee, please include this information.*

*If you are reporting a retrospective study of medical records or archived samples, please ensure that you have discussed whether all data were fully anonymized before you accessed them and/or whether the IRB or ethics committee waived the requirement for informed consent. If patients provided informed written consent to have data from their medical records used in research, please include this information.*

*Once you have amended this/these statement(s) in the Methods section of the manuscript, please add the same text to the “Ethics Statement” field of the submission form (via “Edit Submission”).*

*For additional information about PLOS ONE ethical requirements for human subjects research, please refer to* [*http://journals.plos.org/plosone/s/submission-guidelines#loc-human-subjects-research*](http://journals.plos.org/plosone/s/submission-guidelines#loc-human-subjects-research).

**Response:** We have included the full name of the ethics committee/institutional review board(s) that approved our study. This is a retrospective study of medical records. All data were fully anonymized before we accessed them, and therefore inform consent is not required. We added a statement of ethics at the end of the Material and Method section.

*3. In your Data Availability statement, you have not specified where the minimal data set underlying the results described in your manuscript can be found. PLOS defines a study's minimal data set as the underlying data used to reach the conclusions drawn in the manuscript and any additional data required to replicate the reported study findings in their entirety. All PLOS journals require that the minimal data set be made fully available. For more information about our data policy, please see*

[*http://journals.plos.org/plosone/s/data-availability*](http://journals.plos.org/plosone/s/data-availability)*.*

*"Upon re-submitting your revised manuscript, please upload your study’s minimal underlying data set as either Supporting Information files or to a stable, public repository and include the relevant URLs, DOIs, or accession numbers within your revised cover letter. For a list of acceptable repositories, please see* [*http://journals.plos.org/plosone/s/data-availability#loc-recommended-repositories*](http://journals.plos.org/plosone/s/data-availability#loc-recommended-repositories)*.*

*Any potentially identifying patient information must be fully anonymized.*

*Important: If there are ethical or legal restrictions to sharing your data publicly, please explain these restrictions in detail. Please see our guidelines for more information on what we consider unacceptable restrictions to publicly sharing data: http://journals.plos.org/plosone/s/data-availability#loc-unacceptable-data-access-restrictions. Note that it is not acceptable for the authors to be the sole named individuals responsible for ensuring data access.*

*We will update your Data Availability statement to reflect the information you provide in your cover letter.*

**Response:** Data Availability Statement: our results were compliance with PLOS' data policy.

*4. PLOS requires an ORCID iD for the corresponding author in Editorial Manager on papers submitted after December 6th, 2016. Please ensure that you have an ORCID iD and that it is validated in Editorial Manager. To do this, go to ‘Update my Information’ (in the upper left-hand corner of the main menu), and click on the Fetch/Validate link next to the ORCID field. This will take you to the ORCID site and allow you to create a new iD or authenticate a pre-existing iD in Editorial Manager. Please see the following video for instructions on linking an ORCID iD to your Editorial Manager account: https://www.youtube.com/watch?v=_xcclfuvtxQ.*

**Response:** We attached our ORCID iD for the corresponding author upon paper submission.

Response to Reviewer #1

*The manuscript makes a retrospective study of twin pregnancies in the regional hospital by comparing the vaginal and Cesarean deliveries. This paper is in a good quality to discuss the potential effects of delivery modes on the maternal characteristics as well as the neonatal outcomes.*

*Major comments:*

1. *Are there any other placental parameters collected from these Cesarean deliveries?*

**Response:** Thank you for pointing out this important information. According to chart review, the placenta sites were detected only by ultrasound before delivery. During Cesarean section, the placentae were removed manually, and there were no placenta abnormalities, such as abruptio or velamentus placenta recorded. We added this information on the result section, line 160~161.

*What are the differences between twin A and twin B? Or are they just randomly named? The authors should mention this point.*

**Response:** Twin A was defined as the first newborn delivered and twin B the second newborn. We mentioned this point in our manuscript, line 103-104.

2. *What is the definition of “longer period” in 265?*

**Response:** Thank you for pointing out this ambiguous time period. We cancelled the term “longer period” and make our statement clear. “We did not have follow-up data of these newborns after delivery. All of the newborns sent to NICU returned home uneventfully. The conditions of these newborn after discharged were no longer traced.” (lines 280~281).

*4. The authors have given an excellent discussion for a retrospective study. However, the advantages of this regional hospital in twin pregnancies can be mentioned by combining with the general local data (ie. historical data from Taiwan or Asia), if applied.*

**Response:** We have made the comparison in the first paragraph of our discussion. We wrote: “Our study presents a high vaginal delivery rate of 44.9% in twin pregnancies in a regional teaching hospital in Taiwan. This rate is exceptionally high comparing to data collected from other hospitals in Taiwan, in the Asian countries and even in the world. The vaginal delivery rate in twin pregnancies was reported as 18% in Hong Kong (14), 23-28% in Japan (15), and 25% in the United States (9).” We do not have general local data. Indeed, the majority of twins were delivered by Cesarean section in the University teaching hospital, the National Taiwan University Hospital in Taipei. Residents in National Taiwan university hospital rotated to this study regional hospital for training-ship. They learned different modes of delivery in this regional teaching hospital.

*Minor comments:*

*Please keep a space between the number and the unit. For example, it is suggested to use “<1500 gw” in line 117, and so on.*

**Response:** Thank you for your suggestion. We made the corrections.

Response to Reviewer #2

*There are differences in age, parity and presentation between groups. This poses a problem in comparing the results between the two groups. Although logistic regression analysis was performed, it would be better to compare subgroup analysis (between the age, parity and presentation matched groups).*

**Response:** Thank you for your comment. We performed subgroup analysis according to age, parity and other factors. However, we failed to obtain significant result between the two groups, probably due to limited case number. Therefore, we omitted those results in our manuscript, and stated this as our limitation: “It could be quite informative if we could perform subgroup analysis between the age, parity and presentation matched groups to see possible differences in vaginal delivery from Cesarean delivery. Again, due to limited case number, we failed to obtained significant results.” (lines 274~277)

Response to Reviewer #3

*I must confess: after thoroughly reading this paper a number of times, its potential value chases me. I can foresee this paper as a very interesting contribution, in a field plagued with a considerable lack of evidence. Nonetheless, it has serious drawbacks that preclude its publication in its current form. First of all, the sample size is extremely small for such a large study period (12/13-09/19) in a high reference University Hospital. As being a retrospective cohort, no recruiting data is provided. This, notwithstanding, could be hiding a selection bias. To address this issue I suggest providing further info on the actual amount of twin deliveries at the center during the study period.*

**Results:** We apologize for unclear information. Our hospital is a “regional” teaching hospital, not a University hospital. In the Methods and Materials section, we emphasized this information: “Hsin-Chu Branch of National Taiwan University Hospital, a local teaching hospital” (lines 90~91). We enrolled all cases of twin pregnancy at gestation age ≧ 32 weeks without IUFD of each twin. The total number of deliveries during our study period (12/2013-09/2019) was 5818. And the number of twin delivery was 113, accounted for 1.94% of total delivery. The percentage of twin delivery per year was ranged from 1.65% to 2.17%. There were no selection biases in our study.

*The introduction is correct, well written, and coherent. The materials and methods section drove my attention as it suggests they prepared the data collection in a prospective way. Under such a statement this study becomes more of a cross-sectional study, rather than a cohort, even a retrospective one. In my opinion, the entire section should be re-written coherently with what already happened at the study instead of what they think the editor should read.*

**Results:** Thank you for your comment. We clarified that our study is a retrospective study. In the Methods and Materials section, we wrote: “We reviewed all chart records of patients with viable twin pregnancy at gestational weeks of 32 or above, whom were delivered in Hsin-Chu Branch of National Taiwan University Hospital, a local teaching hospital, from Nov 2013 to Sep 2019. Vaginal or Cesarean deliveries were stated in chart records based on suggestion of obstetrician and patients' choice after comprehensive explanation of risks and benefits, at the outpatient prenatal clinic and during the onset of labor.” (lines 89~95). In addition, we added a flow chart (Figure 1) showing the retrospective steps of the study. In the result section, we rearranged our manuscript accordingly as a retrospective study.

*In the results section, I think the authors devote too much space to explain nonscheduled changes in the fetal presentation at the moment of delivery. Then comes the result that strikes me the most: In the vaginal delivery group TOTAL blood loss was 198 +/- 144 ml, compared to, a far more acceptable value of, 763 +/- 332 ml in the CS group. This is quite an unsustainable finding, dramatically different from those already published by groups studying blood loss during twin births (Whittington JR, et al. AJP Rep. 2020:e330-e334,. Frolova AI, et al Obstet Gynecol. 2016;127:951-956. . Blitz MJ, et al. J Matern Fetal Neonatal Med.;33:3740-3745.). Also, the rate of postpartum hemorrhage in the vaginal delivery group is far too low for being credible unless, again, there is a selection bias underneath.*

**Results:** Thank you for your comment. We believe that the nonscheduled changes in the fetal presentation at the moment of delivery are very important for successful vaginal and cesarean deliveries in twin pregnancy. The young obstetrics need to expect and learn this technique during twin deliveries. We insist not to delict these descriptions in the revised manuscript.

The large difference of estimated blood loss between vaginal delivery and Cesarean delivery was resulted from the including of amniotic fluid as EBL during Cesarean delivery. In our charts review, we found amniotic fluid and blood were recorded as EBL during Cesarean section, and only blood as EBL during vaginal delivery. In vaginal delivery, rupture of membrane occurred before bleeding during placenta delivery. We separated the amniotic fluid while counting EBL. However, in Cesarean section, rupture of membrane, delivery of baby and placenta occurred in very short time. The amniotic fluid and blood were calculated together as EBL. We stated these difference in calculating EBL in our Material and Methods section: “Estimated blood loss (EBL) was calculated from the amount of fluid loss, including blood and amniotic fluid, collected from the evacuation bottles and on the gauzes used. In vaginal delivery, the amniotic fluids were not included as EBL since most amniotic fluid flew out during the procedure of membrane rupture at delivery. (lines 106~110).

*The discussion is interesting and well sustained. However, it is in a strong need of a style revision by a native English speaker. Also, there is an unnecessary allusion to a particular delivery (lines 226-234) that clearly does not belong there.*

**Results:** We have sent our revised MS to a native English speaker for style revision. We wrote detail of a difficult vaginal delivery with longest DTI. In the situation of that case, the obstetrician may choose to convert to Cesarean section. However, an experienced obstetrician could complete the vaginal delivery, and the maternal and neonatal outcomes were acceptable. We keep this allusion to demonstrate the important of obstetric experience for high success rate of vaginal delivery in twin pregnancy.

*The conclusion is far too long and could be summarized in the last paragraph*

**Response:** We have shortened our conclusion as: “Our study provides some suggestions for successful vaginal delivery in twin pregnancy. Presentation of first twin is the major factor of successful vaginal delivery of twin and also an important factor related to Apagar score. With careful case selection and adequate clinical experience, vaginal delivery could be safely performed in twin pregnancies with the additional benefit of less blood loss and better recovery compared with Cesarean delivery.” (lines 290~295)

Response to Reviewer #4

*The manuscript entitled "Experience in Different Modes of Delivery in Twin Pregnancy" by Jackie Jung Chen et al. is a retrospective study, which included the data from a regional hospital in Taiwan. The current manuscript compared the difference in the outcome of 98 pairs of twin pregnancy on vaginal delivery and cesarean section. The study revealed that there is not much difference in outcome when compared between vaginal pregnancy and cesarean section. There are multiple grammatical and spelling errors throughout the manuscript.*

**Response:** We have sent the revised MS to a native English speaker for grammatical and spelling checkup.

We thank you for your comments.
